# Role Of Cgrp In Sensitization Of Dura Mater

**DOI:** 10.1100/tsw.2001.428

**Published:** 2001-11-06

**Authors:** K. Messlinger

**Affiliations:** Department of Physiology and Experimental Pathophysiology, University of Erlangen-Nuernberg, 91054 Erlangen, Germany

## Abstract

The mammalian dura mater encephali is richly supplied by trigeminal nerve fibers, a considerable proportion of which contains calcitonin gene-related peptide (CGRP). As plasma levels of CGRP are increased in some forms of headaches, the question is in which way CGRP is involved in nociceptive mechanisms within the peripheral and the central trigeminovascular system.

